# Supplementary figures and images for: Biologically-informed regional subset analysis with CatBoost for robust tissue-of-origin prediction
Source: PLoS One. 2025 Dec 4;20(12):e0337106. doi: 10.1371/journal.pone.0337106 (PMC12677570; doi:10.1371/journal.pone.0337106)

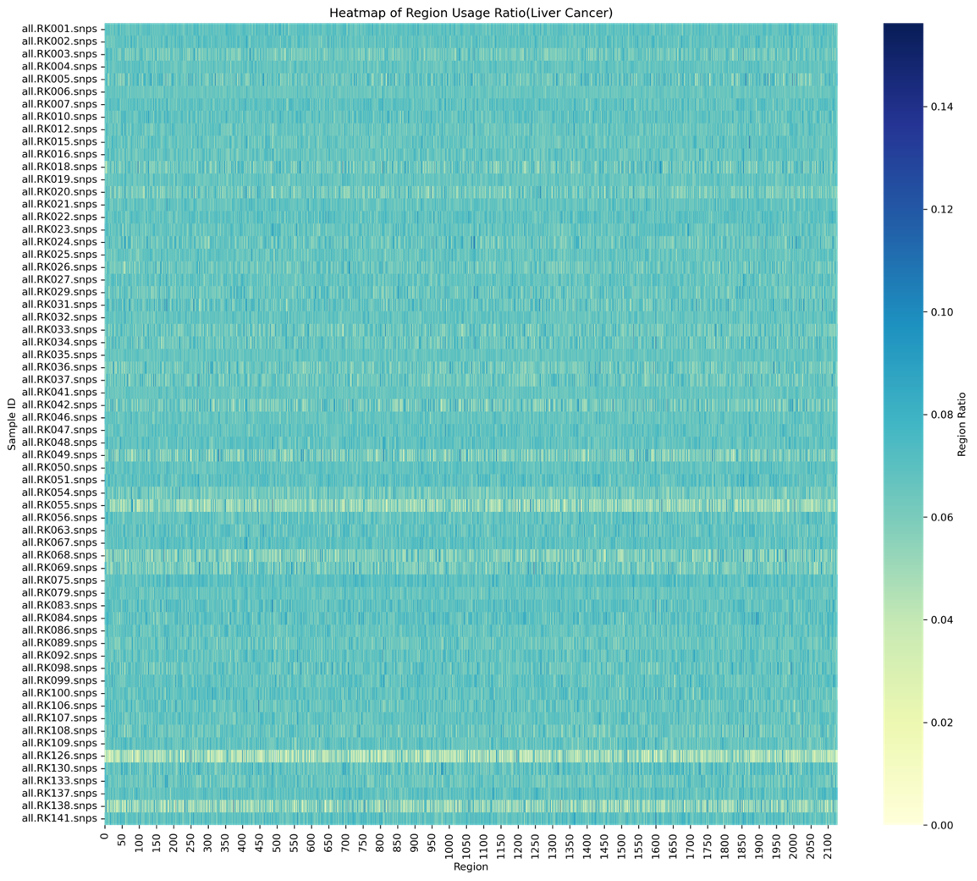

Supplement: S1 Fig — (PNG) [file pone.0337106.s001.png]

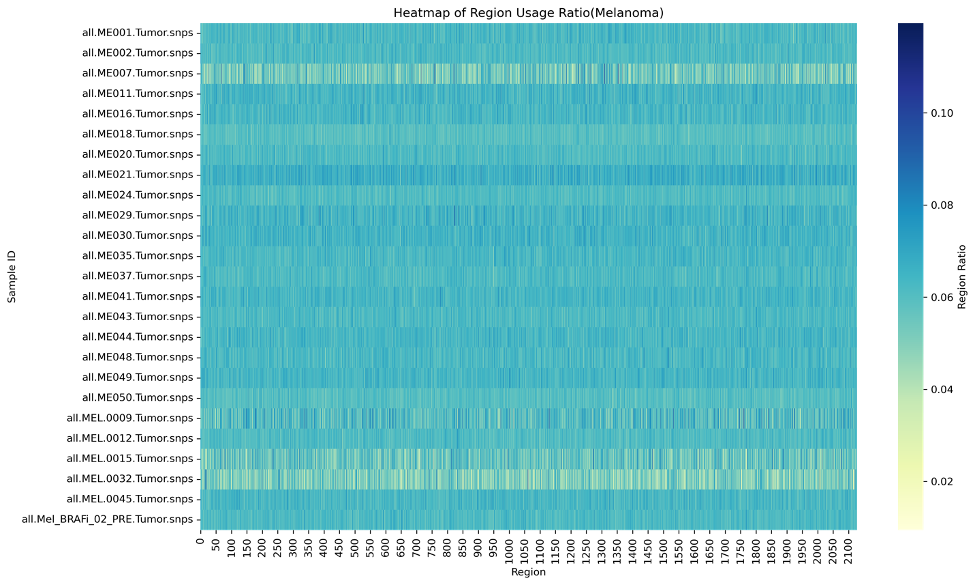

Supplement: S2 Fig — (PNG) [file pone.0337106.s002.png]

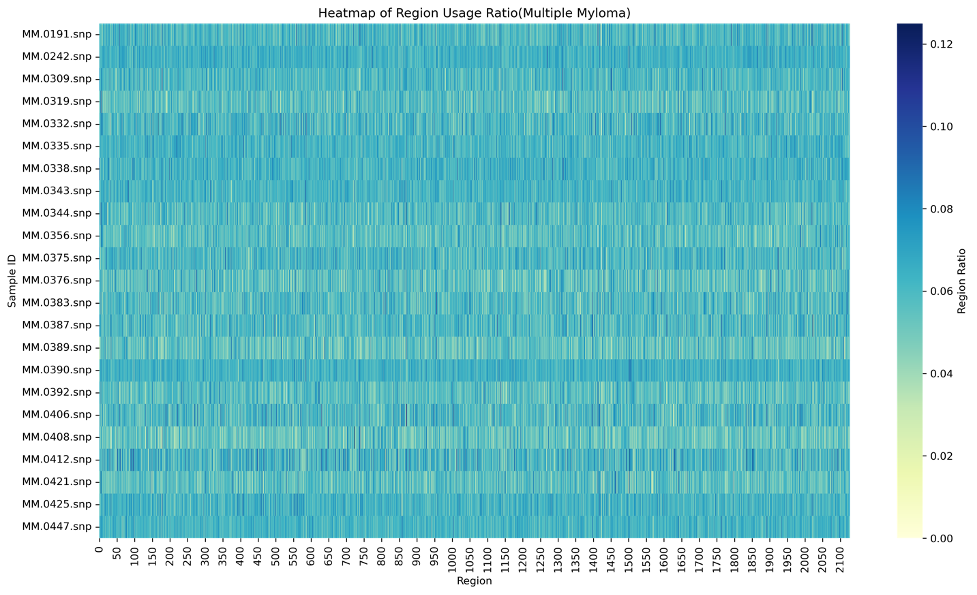

Supplement: S3 Fig — (PNG) [file pone.0337106.s003.png]

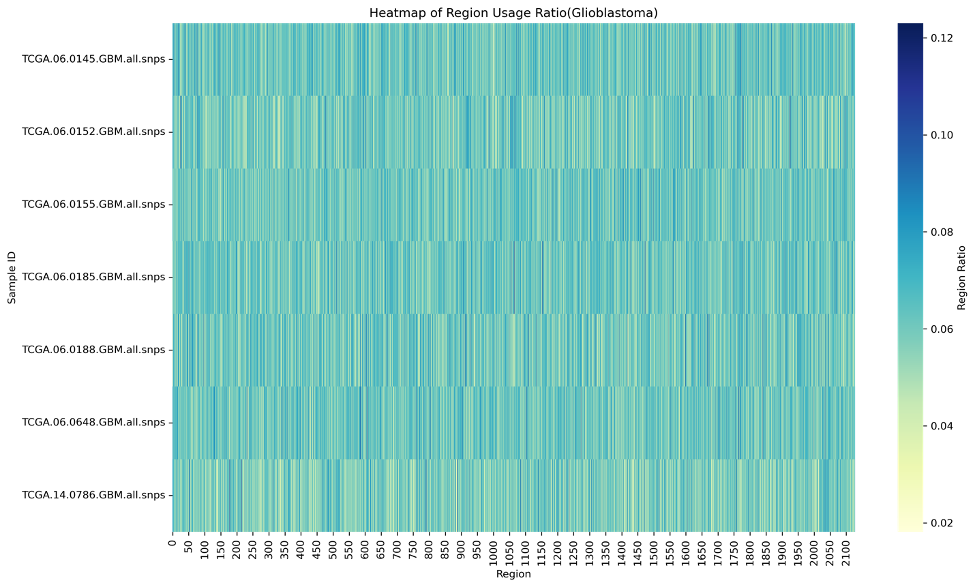

Supplement: S4 Fig — (PNG) [file pone.0337106.s004.png]

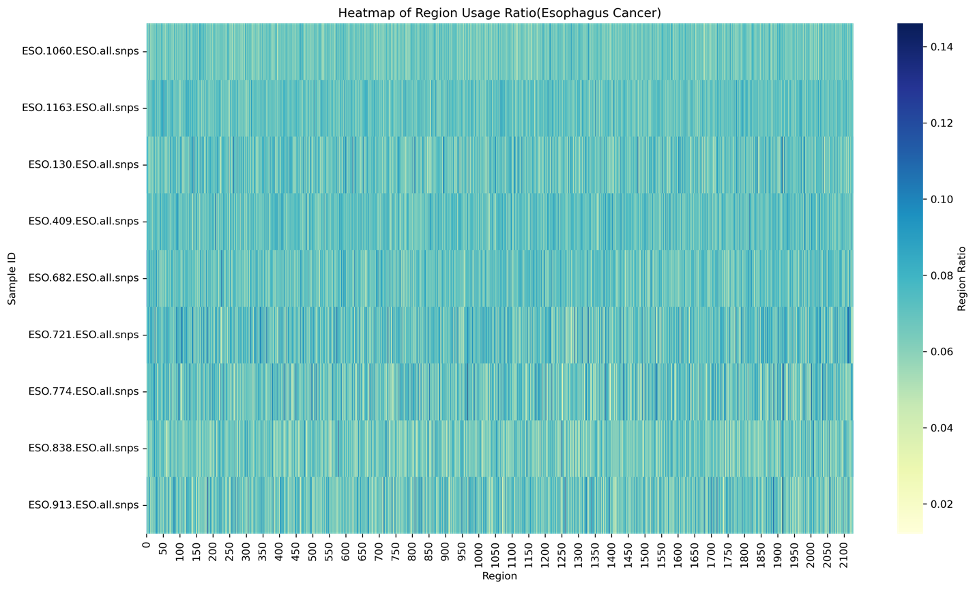

Supplement: S5 Fig — (PNG) [file pone.0337106.s005.png]

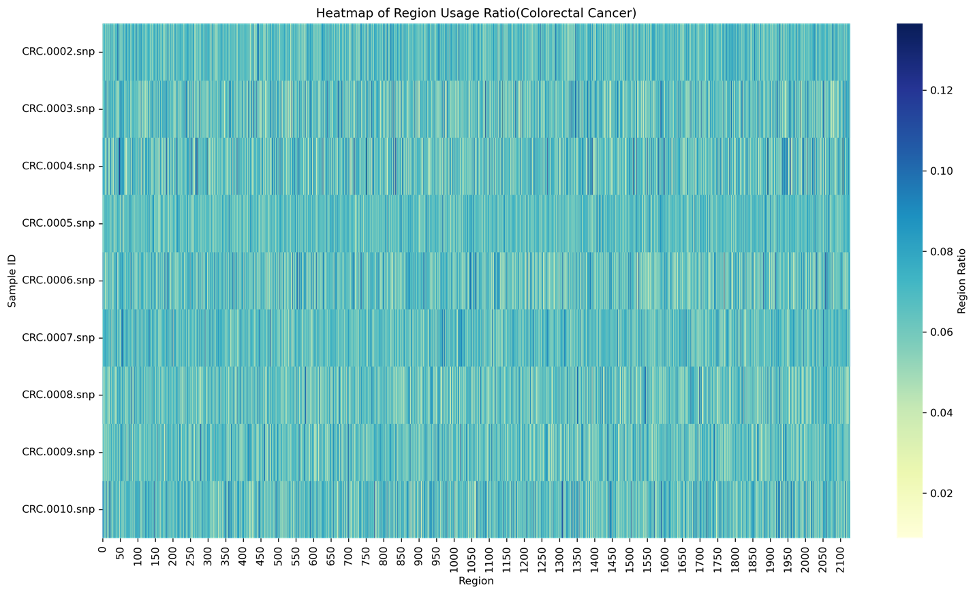

Supplement: S6 Fig — (PNG) [file pone.0337106.s006.png]

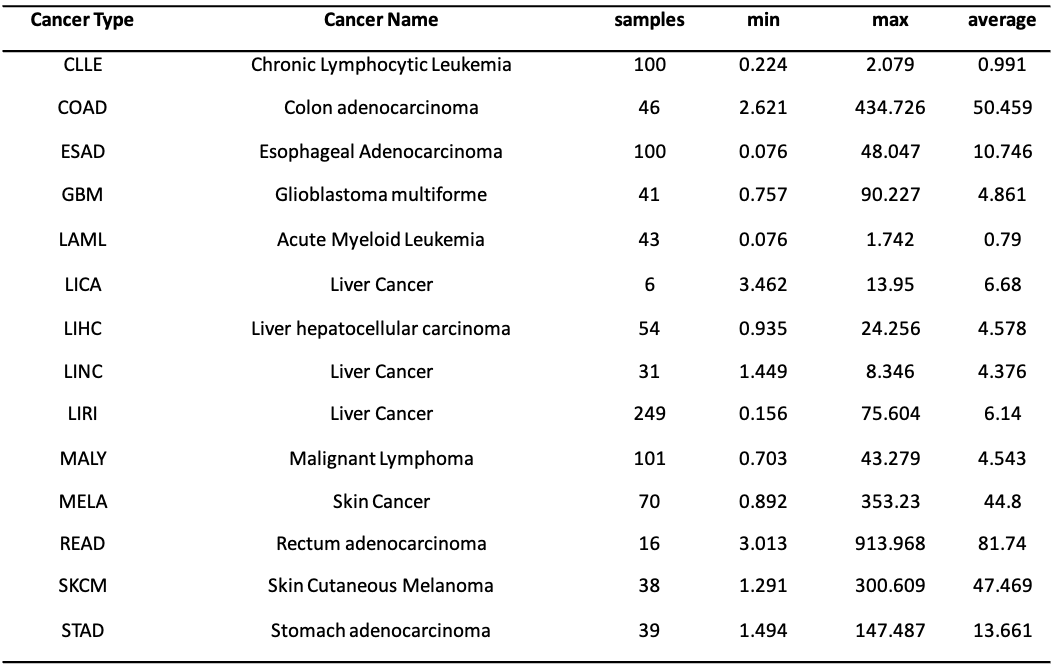

Supplement: S1 Table — (PNG) [file pone.0337106.s007.png]
