# Supplementary material for: Biologically-informed regional subset analysis with CatBoost for robust tissue-of-origin prediction
Source: PLoS One. 2025 Dec 4;20(12):e0337106. doi: 10.1371/journal.pone.0337106 (PMC12677570; doi:10.1371/journal.pone.0337106)
Supplement: Attachment — Submitted filename: Comments PONE-D-25-42292.docx [file pone.0337106.s008.docx]

**Manuscript Number: PONE-D-25-42292**

**Reviewer’s Comments:** The manuscript titled “Biologically-Informed Regional Subset Analysis with CatBoost for Robust Tissue-of-Origin Prediction” presents a CatBoost prediction model for precision oncology and the diagnosis of cancers. This approach achieved a 4% gain in melanoma accuracy and a 4.4% gain in multiple myeloma and perfect (100%) accuracy in high‑mutation cancers such as esophageal adenocarcinoma and glioblastoma with as few as 50 informative regionals subset. On considering all things, these findings show that biologically guided regional subset 168 selection can improve TOO/COO prediction accuracy by lowering input dimensionality, mitigating data sparsity, and capturing important 169 epigenetic–mutation correlations. I am convinced with the method applied for the analysis. However, before publishing, it requires following minor revision to incorporate the all given suggestions.

1. To enhance the comprehensiveness of your paper, it would be beneficial to include at least five previous studies for comparative analysis, thereby strengthening its position relative to existing literature on the same topic.
2. The novelty of the research work is not clear. The authors are suggested to provide suitable novelty aspects.
3. Can you provide justification for claiming that your work over existing work in literature?
4. Labels of Fig. 3 are not clearly visible. Authors need to revise it.
5. Most of the cited reference work is before 2020. It is suggested to add latest relevant references.
