# Supplementary material for: Biologically-informed regional subset analysis with CatBoost for robust tissue-of-origin prediction
Source: PLoS One. 2025 Dec 4;20(12):e0337106. doi: 10.1371/journal.pone.0337106 (PMC12677570; doi:10.1371/journal.pone.0337106)
Supplement: Attachment — Submitted filename: Response to Reviewers.docx [file pone.0337106.s009.docx]

**Manuscript ID:** PONE-D-25-42292
**Title:** *Biologically-Informed Regional Subset Analysis with CatBoost for Robust Tissue-of-Origin Prediction*
**Journal:** PLOS ONE
**Type:** Response to Reviewers
**Date:** 15 Oct, 2025

**Dear Editor and Reviewers,**

We sincerely thank the Academic Editor and the reviewers for their constructive comments and valuable suggestions that helped us improve the quality and clarity of our manuscript.
We have carefully revised the manuscript in accordance with all comments and suggestions. Below, we provide our detailed responses to each point.
All changes have been highlighted in the revised manuscript using track changes.

**Response to Reviewer 1**

**Comment 1:**
*Why authors used the only CatBoost machine learning technique only.*

**Response:**
We thank the reviewer for this important question. In previous studies utilizing tumor mutation and methylation data, algorithms such as Random Forest and XGBoost were commonly applied. These models share the characteristic of being tree-based ensemble methods, primarily aimed at extracting features that can explain model predictions. Moreover, in recent biomedical data analyses, CatBoost has emerged as a widely used interpretable model. Accordingly, we compared the performance of CatBoost with Random Forest and XGBoost in our dataset and observed superior performance. Therefore, we applied our proposed informative regional subset analysis using CatBoost. This explanation has been added to the Introduction section (pages 1, lines 30–36).

**Comment 2:**
*Much more discussion about the results should be given in this paper, especially the author needs to provide enough physical mechanism analysis about the results.*

**Response:**
We appreciate the reviewer’s insightful comment. In response, we have substantially expanded the Discussion section to include not only a description of the common regions identified as important in our analysis across cancer types, but also detailed analyses of their chromosomal locations. Furthermore, we identified the transcripts within these regions using the UCSC Genome Browser and incorporated relevant biological information and our interpretations. These additions provide a more comprehensive discussion of the potential biological mechanisms underlying our findings (Discussion section, pages 11–12, lines 243–297).

**Comment 3:**
*The authors should clarify the mathematical framework used to the CatBoost model.*

**Response:**
Following the reviewer’s suggestion, we have added a detailed explanation of the mathematical framework underlying the CatBoost model. This includes the fundamental principles of gradient boosting and the base model formulation. This new subsection has been incorporated into the Methods section of the revised manuscript. (pages 3–4, lines 120–144)

**Comment 4:**
*The figures in the manuscript are blurred and need improvement.*

**Response:**
We have replaced all blurred figures with high-resolution versions and ensured that the axis labels, legends, and annotations are clearly visible in the revised manuscript. In particular, Figure 3, which appeared noticeably less clear than the others, has been replaced with a high-resolution version to improve visual quality. (Result section, page 8, Figure 3)

**Comment 5:** *In the manuscript, there are grammatical and spelling mistakes. The language is comprehensive and coherent, but there are some mistakes.*

**Response:**
We have carefully proofread the entire manuscript and corrected all grammatical and typographical errors. The revised text has also been checked by a professional English editing service.

**Response to Reviewer 2**

**Comment 1:**
*To enhance the comprehensiveness of your paper, it would be beneficial to include at least five previous studies for comparative analysis, thereby strengthening its position relative to existing literature on the same topic.*

**Response:**
We thank the reviewer for this important suggestion. To enhance the comprehensiveness of our manuscript, we have now compared the datasets, analysis methods, and results of recent related studies. We also summarized the limitations of prior work to highlight the novelty and necessity of our study. These revisions have been added to the Introduction section (pages 1–2, lines 30–63).

**Comment 2:**
*The novelty of the research work is not clear. The authors are suggested to provide suitable novelty aspects.*

**Response:**
We thank the reviewer for this constructive comment. To clarify the novelty of our study, we have explicitly highlighted the main contributions of our work, including a concise description of the novel aspects of our approach and an expanded literature review to contextualize our findings relative to existing studies. These revisions are included in the Introduction section (pages 2, lines 64–88). (pages 2, lines 64–88)

**Comment 3:**
*Can you provide justification for claiming that your work over existing work in literature?*

**Response:**
We appreciate the reviewer’s suggestion. To emphasize the advantages of our study over existing work, we have added a discussion highlighting the limitations of previous studies and the biological and computational strengths of our approach in the Introduction section.(pages 2, lines 48–63)

**Comment 4:**
*Labels of Fig. 3 are not clearly visible. Authors need to revise it.*

**Response:**
We have revised Figure 3 to improve label clarity, font size, and contrast for better readability. (Result section, page 8, Figure 3)

**Comment 5:**
*Most of the cited reference work is before 2020. It is suggested to add latest relevant references.*

**Response:**
We thank the reviewer for this valuable suggestion. In response, we have added more than 10 recent relevant studies to the manuscript. These additions help to highlight the novelty of our work, differentiate it from existing studies, and clarify the limitations of prior research, thereby emphasizing the advantages of our approach. These revisions are included in the Introduction section. (pages 1–2, lines 30–63)

**Conclusion**

We thank the reviewers and editor once again for their time and constructive feedback.
We believe that the revised version has been significantly improved and hope that it now meets PLOS ONE’s publication standards.

Sincerely,
**[Sungmin Yang / Professor Hong-Gee Kim]**
